# Supplementary material for: Identification of Freezing-Responsive microRNAs and Their Targets in Chinese Jujube by Small RNA and Degradome Sequencing
Source: Int J Mol Sci. 2026 Sep 4;27(17):7912. doi: 10.3390/ijms27177912 (PMC13566890; doi:10.3390/ijms27177912)
Supplement: Supplementary file 1 [file ijms-27-07912-s001.zip › Supplementary Material S2/novel_miR_1.pdf]

Provisional ID	:	novel_miR_1
Score total	:	7168.2
Score for star read(s)	:	3.9
Score for read counts	:	7159.1
Score for mfe	:	3.6
Score for randfold	:	1.6
Score for cons. seed	:	
Total read count	:	14054
Mature read count	:	9107
Loop read count	:	31
Star read count	:	4916

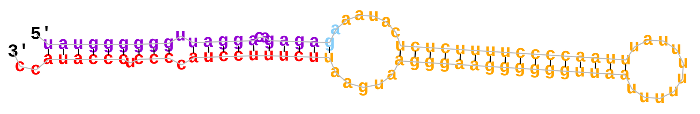

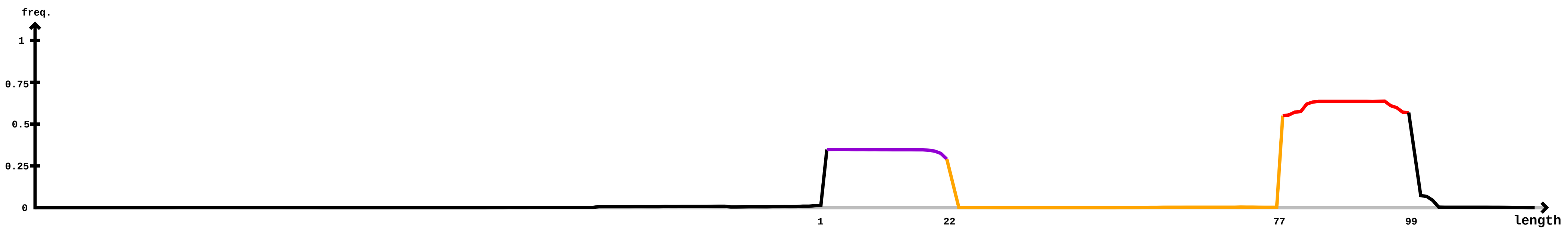

Star Mature

```
5'- uuccuuuuuuucaccaaaaucuagacucaauuaauuucucuuuucccuuccuccaccaucugguguauuucuuuucucacuaaaaggauggaagagggaaaggcuuaagagugugggugg aagugauagguauggggguuaggacagagagaaauacucucuuuuccccaauuuauuuuuuuuaauugggggggaagggaaugaauucuuuccuaccccucccauaccauucauuuccauccuuuccuuc -3' obs
    uuccuuuuuuucaccaaaaucuagacucaauuaauuucucuuuucccuuccuccaccaucugguguauuucuuuucucacuaaaaggauggaagagggaaaggcuuaagaguguggguggaagugauagguauggggguuaggacagagagaaauacucucuuuuccccaauuuauuuuuuuuaauugggggggaagggaaugaauucuuuccuaccccucccauaccauucauuuccauccuuuccuuc       exp
    ....................................(((((((((..((((((...(((((...........)))))...)).)))).))))))))))...(((.(...((((((((((((..((((((((((((.(((((..(((((......(((((((((((((((..........)))))))))))))))).....)))))))))).))).))))))))..))))))))))))...)))).
```

read	reads	mm	sample
caccaaaaucuagacucaauu	1	0	LT-1
ccaaaaucuagacucaauuaauuucucu	1	0	JCK-2
aaucuagacucaauuaauuucuc	1	0	LT-3
uagacucaauuaauuucucuuuuc	1	0	JCK-1
agacucaauuaauuucucuuuucc	1	0	JCK-3
agacucaauuaauuucucuuuucc	1	0	JCK-1
agacucaauuaauuucucuuuucc	1	0	JCK-2
ucugguguauuucuuuucuc	2	0	JCK-3
ucucacuaaaaggauggaagagggaa	2	0	LT-1
ucucacuaaaaggauggaagagggaaagg	1	0	JCK-3
ucucacuaaaaggauggaagagggaaagg	1	0	JCK-1
cucacuaaaaggauggaagagggaaagg	1	0	JCK-3
cacuaaaaggauggaagagg	1	0	LT-3
cacuaaaaggauggaagagggaaagg	1	0	JCK-2
cacuaaaaggauggaagagggaaaggA	2	1	JCK-2
cacuaaaaggauggaagagggaaaggA	2	1	JCK-3
aaaaggauggaagagggaaagg	1	0	LT-3
aaaaggauggaagagggaaagg	4	0	LCK-3
aaaggauggaagagggaaagg	1	0	LT-3
aggauggaagagggaaagg	3	0	LT-2
agagggaaaggcuuaagagu	5	0	LT-2
agagggaaaggcuuaagagug	1	0	LT-3
agagggaaaggcuuaagagugu	36	0	LCK-2
agagggaaaggcuuaagagugu	21	0	LCK-1
gagggaaaggcuuaagagug	1	0	JCK-2
Cgggaaaggcuuaagagugugg	1	1	LT-1
aaaggcuuaagagugugggugg	1	0	LT-3
aaaggcuuaagaguguggguggaagu	2	0	JCK-1
aggcuuaagagugugggug	1	0	LT-1
cuuaagaguguggguggaaguga	2	0	JT-3
cuuaagaguguggguggaaguga	2	0	JT-2
cuuaagaguguggguggaagugauag	2	0	JCK-3
cuuaagaguguggguggaagugauag	2	0	JCK-2

Star Mature

```
uuccuuuuuuucaccaaaaucuagacucaauuaauuucucuuuucccuuccuccaccaucugguguauuucuuuucucacuaaaaggauggaagagggaaaggcuuaagaguguggguggaagugauagguaugggggguuaggacagagagaaauacucucuuuuccccaauuuauuuuuuuuuaauuggggggaagggaaugaauucuuuccuaccccucccauaccauucauuuccauccuuuccuuc
.......................................................................................................cuuaagaguguggguggaagugauagg.........................................................................................................................  1  0  JCK-3
.......................................................................................................cuuaagaguguggguggaagugauagg.........................................................................................................................  1  0  LT-2
.......................................................................................................cuuaagaguguggguggaagugauagg.........................................................................................................................  5  0  JCK-2
.......................................................................................................cuuaagaguguggguggaagugauagg.........................................................................................................................  3  0  JT-3
.......................................................................................................cuuaagaguguggguggaagugauagg.........................................................................................................................  2  0  LT-1
.......................................................................................................cuuaagaguguggguggaagugauagg.........................................................................................................................  4  0  JT-2
..........................................................................................................aagaguguggguggaagugauagg.........................................................................................................................  4  0  JT-3
..........................................................................................................aagaguguggguggaagugauagg.........................................................................................................................  2  0  JT-2
..............................................................................................................guguggguggaagugauagg.........................................................................................................................  1  0  JCK-3
..............................................................................................................guguggguggaagugauagg.........................................................................................................................  1  0  JCK-1
...............................................................................................................uguggguggaagugauaggu........................................................................................................................  10  0  LCK-3
................................................................................................................guggguggaagugauagguau......................................................................................................................  1  0  LT-1
................................................................................................................guggguggaagugauagguaug.....................................................................................................................  8  0  LCK-3
.................................................................................................................ugggugg aagugauagguaugg....................................................................................................................  2  0  JCK-3
...................................................................................................................gguggaagugauagguauggg...................................................................................................................  1  0  LT-3
....................................................................................................................guggaagugauagguaug.....................................................................................................................  5  0  LCK-3
....................................................................................................................guggaagugauagguauggg...................................................................................................................  1  0  LT-1
....................................................................................................................guggaagugauagguaugggg..................................................................................................................  2  0  JCK-2
....................................................................................................................guggaagugauagguaugggg..................................................................................................................  1  0  JCK-3
.....................................................................................................................uggaagugauagguaugg.....................................................................................................................  1  0  LT-3
.....................................................................................................................uggaagugauagguaugggg..................................................................................................................  2  0  JCK-2
.....................................................................................................................uggaagugauagguaugggg..................................................................................................................  1  0  JCK-3
.....................................................................................................................uggaagugauagguaugggggg................................................................................................................  6  0  LCK-3
.........................................................................................................................agugauagguaugggggguu..............................................................................................................  3  0  LT-3
.........................................................................................................................agugauagguaugggggguuaggaca........................................................................................................  7  0  LCK-3
.........................................................................................................................agugauagguaugggggguuaggacagaga....................................................................................................  2  0  LCK-3
...........................................................................................................................ugauagguaugggggguuaggaca........................................................................................................  3  0  LT-3
.............................................................................................................................auagguaugggggguuaggacaga......................................................................................................  1  0  JCK-2
.............................................................................................................................auagguaugggggguuaggacaga......................................................................................................  1  0  JCK-1
..............................................................................................................................uagguaugggggguuagga..........................................................................................................  1  0  JCK-1
..............................................................................................................................uagguaugggggguuagga..........................................................................................................  4  0  LT-3
..............................................................................................................................uagguaugggggguuaggac.........................................................................................................  2  0  JCK-2
..............................................................................................................................uagguaugggggguuaggacU........................................................................................................  1  1  LT-3
..............................................................................................................................uagguaugggggguuaggacC........................................................................................................  2  1  JCK-3
..............................................................................................................................uagguaugggggguuaggaca........................................................................................................  6  0  JCK-3
..............................................................................................................................uagguaugggggguuaggaca........................................................................................................  3  0  JT-3
..............................................................................................................................uagguaugggggguuaggaca........................................................................................................  1  0  JCK-1
..............................................................................................................................uagguaugggggguuaggaca........................................................................................................  3  0  LT-1
..............................................................................................................................uagguaugggggAuuaggaca........................................................................................................  1  1  LT-1
..............................................................................................................................uagguaugggggguuaggaca........................................................................................................  6  0  JCK-2
..............................................................................................................................uagguaugggggguuaggaca........................................................................................................  1  0  JT-2
..............................................................................................................................uagguaugggggguuaggacC........................................................................................................  1  1  LT-3
..............................................................................................................................uagguaugggggguuaggaca........................................................................................................  4  0  LCK-3
..............................................................................................................................uagguaugggggguuaggaca........................................................................................................  1  0  LT-3
..............................................................................................................................uagguaugggggguuaggacaga......................................................................................................  1  0  JCK-3
................................................................................................................................gguaugggggguuaggacag.......................................................................................................  1  0  JCK-3
................................................................................................................................gguaugggggguuaggacaga......................................................................................................  1  0  LCK-3
................................................................................................................................gguaugggggguuaggacaga......................................................................................................  9  0  JCK-1
................................................................................................................................gguaugggggguuaggacaga......................................................................................................  3  0  JCK-2
................................................................................................................................gguaugggggguuaggacaga......................................................................................................  4  0  LT-1
................................................................................................................................gguaugggggguuaggacaga......................................................................................................  12  0  JT-1
................................................................................................................................gguaugggggguuaggacaga......................................................................................................  3  0  LT-3
................................................................................................................................gguaugggggguuaggacaga......................................................................................................  9  0  LT-2
................................................................................................................................gguaugggggguuaggacaga......................................................................................................  4  0  JCK-3
.................................................................................................................................guaugggggguuaggacaga......................................................................................................  1  0  LT-3
.................................................................................................................................guaugggggguuaggacaga......................................................................................................  2  0  LT-1
.................................................................................................................................guaugggggguuaggacaga......................................................................................................  6  0  LCK-3
.................................................................................................................................guaugggggguuaggacagag.....................................................................................................  1  0  JCK-3
.................................................................................................................................Cuaugggggguuaggacagaga....................................................................................................  2  1  JCK-2
.................................................................................................................................Cuaugggggguuaggacagaga....................................................................................................  2  1  JCK-1
.................................................................................................................................Cuaugggggguuaggacagaga....................................................................................................  1  1  LT-3
.................................................................................................................................Cuaugggggguuaggacagaga....................................................................................................  7  1  JT-1
..................................................................................................................................uaugggggguuaggacag.......................................................................................................  18  0  JCK-3
..................................................................................................................................uaugggggguuaggacag.......................................................................................................  10  0  LT-3
..................................................................................................................................uaugggggguuCggacag.......................................................................................................  1  1  JCK-2
..................................................................................................................................uaugggggguuaggacag.......................................................................................................  8  0  LT-1
..................................................................................................................................uaugggggguuaggacag.......................................................................................................  10  0  LCK-3
..................................................................................................................................uaugggggguuaggacag.......................................................................................................  9  0  JCK-1
..................................................................................................................................uaugggggguuaggacag.......................................................................................................  8  0  LT-2
..................................................................................................................................uaugggggguuaggacag.......................................................................................................  14  0  JCK-2
```

Star Mature

uuccuuuuuuucaccaaaaucuagacucaauuaauuucucuuuucccuuccuccaccaucugguguauuucuuuucucacuaaaaggauggaagagggaaaggcuuaagagugugggguggaagugauagguaugggggguuaggacagagagaaauacucucuuuuccccaauuuauuuuuuuuaauuggggggaagggaaugaauucuuuccuaccccucccauaccauucauuuccauccuuuccuuc

uaugggggguuaggacaga	14	0	LT-3
uaugggggguuaggacaga	8	0	LT-1
uaugggggguuaggacaga	37	0	LCK-3
uaugggggguuaggacaga	11	0	JT-2
uaugggggguuaggacaga	5	0	JT-3
uaugggggguuaggacaga	11	0	JT-1
uaugggggguuaggacaga	19	0	LT-2
uCugggggguuaggacaga	1	1	JCK-1
uaugggggguuaggacagU	1	1	JCK-2
uaugggggguuaggacagU	1	1	LT-1
uaugggggguuaggacaga	12	0	JCK-3
uNugggggguuaggacaga	1	1	JT-2
uaugggggguuaggacaga	11	0	JCK-2
uaugggggguuaggacagag	34	0	LT-1
uCugggggguuaggacagag	1	1	JT-3
uaugggggguuCggacagag	1	1	JCK-2
uaugggggguuaggacagag	60	0	JCK-1
uaugggggguuaggacagag	16	0	LCK-3
uaugggggguuaggacagaN	1	1	JCK-1
uCugggggguuaggacagag	3	1	JCK-2
uaugggggguuaggacagag	62	0	JCK-3
uaugggggguuaggacagag	115	0	JT-1
uaugggggguuaggacagag	7	0	JT-2
uaugggggguuaggacagag	91	0	JCK-2
uaugggggguuaggacagag	35	0	LT-2
uaugggggguuaggacagag	20	0	JT-3
uaugggggguuaggacagag	43	0	LT-3
uaugggggguuNggacagag	1	1	JCK-1
uaugggggguuaggacagaU	1	1	LT-2
uauAggggguuaggacagaga	1	1	JCK-3
uaugggggguuagAacagaga	1	1	LCK-3
uaugggggguuaggacagaga	113	0	LCK-1
uaugggggguuaggacagaga	345	0	LCK-3
uaNgggggguuaggacagaga	1	1	LCK-2
uauAggggguuaggacagaga	1	1	JCK-1
uCugggggguuaggacagaga	6	1	JCK-2
uaugggggguuaggacagagC	2	1	JCK-3
uCugggggguuaggacagaga	3	1	JT-1
uaugggggguuCggacagaga	1	1	JCK-3
Caugggggguuaggacagaga	1	1	LT-2
uaugggggguuaggCcagaga	1	1	JT-2
uauAggggguuaggacagaga	1	1	JT-1
uaugggggguuaggacagaga	88	0	LCK-2
uNugggggguuaggacagaga	1	1	JCK-2
uaugggggguuaggacagCga	1	1	LT-2
uauggggggCuaggacagaga	1	1	JT-2
uaugggggguuNggacagaga	1	1	JCK-1
uCugggggguuaggacagaga	4	1	LT-2
uaugggggguuaggCcagaga	1	1	LT-3
uaugggggguuaggacagaga	231	0	JT-3
uaugggggguuaggacagaga	192	0	JT-2
uaugggggguuaggacagaga	589	0	JT-1
uaugggggguuaggCcagaga	2	1	JCK-2
uauAggggguuaggacagaga	1	1	JCK-2
uaugggggguuaggacagaNa	1	1	JCK-1
uaugggggguuaggCcagaga	1	1	JCK-1
uaCgggggguuaggacagaga	1	1	JT-3
uaugggUgguuaggacagaga	1	1	JT-3
uaugggggguuaggacagagU	1	1	JCK-1
uaugggggguuaggacCgaga	1	1	JCK-2
uaugggggguuaggacagaga	206	0	LT-1
uaugggggguuaggacagaga	289	0	LT-3
uaugggggguuagNacagaga	2	1	LCK-1
uaugggggguuaggacagagC	2	1	JCK-1
uaugggggguuaggCcagaga	1	1	JT-1
uaugggggguuaggacagaga	478	0	JCK-1
uaugggggguuagUacagaga	1	1	LCK-2
uaugggggguuaggCcagaga	1	1	JCK-3
uaugggggguuaggacagaga	242	0	LT-2
uaugggggguuaggCcagaga	1	1	LCK-1

Star **Mature**

uuccuuuuuuucaccaaaaucuagacucaauuaauuucucuuuucccuuccuccaccaucugguguauuucuuuucucacuaaaaggauggaagagggaaaggcuuaagagugugggguggaagugauagguaugggggguuaggacagagagaaauacucucuuuccccaauuuauuuuuuuuaauuggggggaagggaaugaauucuuuccuaccccucccauaccauucauuuccauccuuuccuuc

uaugggggguuaggacagaga	727	0	JCK-2
uaugggggguuCggacagaga	1	1	LCK-2
uaugggggguuaggacagaga	555	0	JCK-3
uaugggggguuaggacagagU	1	1	LT-1
uCugggggguuaggacagaga	1	1	JCK-3
uCugggggguuaggacagaga	1	1	JT-3
uaugggggguuCggacagaga	1	1	LCK-3
uaugggggguuaggacagagU	3	1	LT-3
uCugggggguuaggacagaga	2	1	LCK-3
uaugggggguuaggacagagaU	1	1	JCK-2
uaugggggguuaggacagagaU	1	1	LT-3
uaugggggguuaggacagagaC	3	1	JCK-2
uaugggggguuaggacagagaU	2	1	LT-1
uaugggggguuaggacagagaU	1	1	LT-2
uaugggggguuaggacagagaC	1	1	JCK-3
uaugggggguuaggacagagagaa	2	0	JCK-2
augggggguuaggacaga	2	0	LT-2
augggggguuaggacaga	1	0	LT-3
augggggguuaggacagag	1	0	LT-2
augggggguuaggacagag	3	0	JCK-1
augggggguuaggacagag	1	0	JCK-2
augggggguuaggacagaga	5	0	JCK-3
augggggguuaggacagaga	2	0	JCK-2
augggggguuaggacagagaU	1	1	JCK-2
ugggggguuaggacagaga	3	0	JCK-2
ugggggguuaggacagaga	1	0	JCK-1
ggggguuaggacagagagaaaua	1	0	LT-3
gggguuaggacagagagaaauac	1	0	LT-3
ggguuaggacagagagaaauac	4	0	LCK-3
guuaggacagagagaaauacu	3	0	LCK-3
acucucuuuccccaauuuUuuuuuu	1	1	LT-1
ucucuuuccccaauuuauA	1	1	LT-3
uuuuuaauuggggggaaggga	5	0	LCK-3
uuuuuaCuuggggggaaggga	1	1	LCK-3
uuaauuggggggaagggaau	1	0	LT-3
uuaauuggggggaagggaaug	1	0	LT-1
uuaauuggggggaCgggaaug	1	1	LT-1
uaauuggggggaagggaaugaau	1	0	LT-3
uaauuggggggaagggaaugaauA	6	1	LCK-3
uaauuggggggaagggaaugaauuc	2	0	LCK-3
uaauuggggggaagggaaugaauuc	2	0	LT-3
aauuggggggaagggaau	6	0	LCK-3
uuggggggaagggaaugaauucuuuc	7	0	LCK-3
uggggggaagggaaugaauucu	1	0	JCK-2
uggggggaagggaaugaauucu	1	0	JCK-1
ggggaagggaaugaauucu	1	0	LT-1
aaugaauucuuuccuaccccuc	7	0	JT-3
aaugaauucuuuccuaccccuc	4	0	JT-2
Cucuuuccuaccccuccca	1	1	LT-1
ucuuuccuaccccuccca	35	0	LCK-2
ucuuuccuaccccuccca	3	0	JT-1
ucuuuccuaccccuccca	22	0	JCK-2
ucuuuccuaccccNccca	1	1	LCK-1
ucuuCccuaccccuccca	1	1	LT-2
ucuuuccuaccccuccca	9	0	JT-2
ucuuuccuaccccuccca	50	0	LT-3
ucuuuccuUccccuccca	3	1	LT-2
ucuuuccuacAccuccca	1	1	LCK-3
ucuuuccuaccccuccca	67	0	LCK-3
ucuuuccuaccccuccca	16	0	JCK-1
ucuuuccuaccccucccU	1	1	LCK-3
ucuuuccuaccccuccca	9	0	JT-3
ucuuuccuaccccuccca	40	0	LT-1
ucuuuccuaccccuccca	88	0	LT-2
ucuuuccuaccccuccca	33	0	LCK-1
ucuuuccuaccccuccca	9	0	JCK-3
ucuuuccuaccccucccau	14	0	JCK-2
ucuuuccuaccccuGccau	1	1	LCK-1
ucuuuccuaccccucccau	14	0	LT-2
ucuuuccuaccccucccau	12	0	JCK-1

Star Mature

uuccuuuuuuucaccaaaaucuagacucaauuaauuucucuuuucccuuccuccaccaucugguguauuucuuuucucacuaaaaggauggaagagggaaaggcuuaagaguguggguggaagugauaggaugggggguuaggacagagagaaauacucucuuuuccccaauuuauuuuuuuuaauugggggggaagggaaugaauucuuuccuaccccucccauaccauucauuuccauccuuuccuuc

ucuuuccuaccccuAccau	1	1	LCK-3
ucuuuccuaccccucccau	27	0	LCK-1
ucuuuccuaccccucccau	6	0	JCK-3
ucuuuccuaccccucccau	25	0	LT-3
ucuuuccuaccccucccau	26	0	LCK-2
ucuuuccuaccccucccau	17	0	LT-1
ucuuuccuaccccucccau	23	0	LCK-3
ucuuuccuaccccucccauC	2	1	LT-3
ucuuuccuaccccucccaua	53	0	LCK-3
ucuuuccuaccccucccaua	59	0	JCK-2
ucuuuccuaccccucccauU	2	1	LT-3
ucuuuccuaccccucccaua	41	0	LT-2
uNuuuccuaccccucccaua	1	1	JCK-2
ucuuuccuaccccucccCua	1	1	LT-3
ucuuuccuaccccucccauU	2	1	LT-1
ucuuuccuaccccucccaua	57	0	JCK-3
ucuuuccuaccccucccaAa	1	1	JCK-3
ucuuuccuaccccucccaua	35	0	LT-3
ucuuuccuacUccucccaua	28	1	JT-1
ucuuuccuaccccucccaua	42	0	LT-1
ucuuuccuaccccucccaua	43	0	JCK-1
ucuuuccuaccccucccauac	5	0	JCK-3
ucuuuccuaccccucccauaA	1	1	LT-1
ucuuuccuaccccucccauac	4	0	LT-3
ucuuuccuaccccucccauac	8	0	LT-2
ucuuuccuaccccucccauac	7	0	JCK-2
ucuuuccuaccccucccauac	1	0	JCK-1
ucuuuccuaccccucccauac	3	0	LT-1
ucuuuccuCcccucccauacc	7	1	JCK-2
uNuuuccuaccccucccauacc	1	1	JCK-3
ucuuuccuaccccucccauacU	2	1	JCK-1
ucuuuccuaccccucccauCcc	1	1	LT-1
ucuGuccuaccccucccauacc	1	1	JCK-1
ucuuuccuacUccucccauacc	80	1	LCK-2
Ccuuuccuaccccucccauacc	1	1	JCK-3
uNuuuccuaccccucccauacc	1	1	JCK-1
uNuuuccuaccccucccauacc	4	1	JCK-2
ucuCuccuaccccucccauacc	1	1	LT-3
uUuuuccuaccccucccauacc	1	1	JCK-1
ucuuuccuaccccuUccauacc	1	1	JCK-3
ucuuuccuaccccuccUauacc	2	1	LT-3
ucuuuccuaccccucccauaUc	1	1	JCK-2
ucuuuccuaccccucccauacc	214	0	LCK-3
ucuuuccuaUcccucccauacc	3	1	LT-3
ucuuucUuaccccucccauacc	3	1	JCK-2
ucuuuccuaccccuUccauacc	4	1	JCK-1
ucuuCccuaccccucccauacc	1	1	JCK-3
ucuuuUcuaccccucccauacc	1	1	JCK-1
ucuuuccuacUccucccauacc	26	1	JT-3
ucuuuccuCcccucccauacc	1	1	LT-1
ucuuuccuaccccucccauacc	419	0	LT-3
ucCuuccuaccccucccauacc	1	1	LT-2
ucuuuccuaccccucccCuacc	1	1	LT-2
ucuuuccuacGccucccauacc	2	1	JCK-1
ucuuuccuacccAucccauacc	1	1	LCK-3
ucuuuccuaccccucccauaGc	1	1	JCK-2
ucuuuccuaccccucccauacA	1	1	JCK-2
Ccuuuccuaccccucccauacc	2	1	LT-2
ucuuuccuaccccucccCuacc	2	1	JCK-3
ucuuuccuaccccucccauaUc	1	1	JCK-3
uUuuuccuaccccucccauacc	3	1	JCK-2
ucCuuccuaccccucccauacc	1	1	JCK-1
ucuuuccuaccccucccauaGc	1	1	JCK-1
ucuuuccuaccccucccauacc	18	0	LCK-2
ucuuuccuaccccucccauCcc	2	1	LT-3
ucuuuccuaccccucccauCcc	1	1	LT-2
ucuuuccuaAcccucccauacc	2	1	JCK-1
ucCuuccuaccccucccauacc	1	1	JCK-3
ucuuuccuCcccucccauacc	2	1	LT-3
ucuuuccuacUccucccauacc	14	1	JT-2

Star	Mature			
uuccuuuuuuucaccaaaaucuagacucaauuaauuucucuuuucccuuccuccaccaucugguguauuucuuuucucacuaaaaggauggaagagggaaaggcuuaagagugugggugggaagugauagguauggggguuaggacagagagaaauacucucuuuuccccaauuuauuuuuuuuaauugggggaagggaaugaauucuuuccuaccccucccauaccauucauuuccauccuuuccuuc				
ucuuuccuacUccucccauacc		87	1	LCK-1
ucuuuccuaccccucccCuacc		4	1	JCK-1
ucuuuccuaccccucccauacc		434	0	LT-1
ucuuuccuaccccucccauNcc		1	1	JCK-1
ucuuuccuaccccCcccauacc		1	1	JCK-2
ucuuuccuaccccucccauCcc		3	1	JCK-2
ucuuuccuaccccucccauacc		29	0	JT-2
ucuuuccuCccccucccauacc		3	1	JCK-3
ucCuuccuaccccucccauacc		1	1	JCK-2
ucuuuccuaccccucccauacc		48	0	JT-1
Ccuuuccuaccccucccauacc		3	1	JCK-2
ucuuuUcuaccccucccauacc		4	1	JCK-2
ucuuuccuaccccucUcauacc		1	1	LCK-3
ucuuuccuaccccucccauacc		1146	0	JCK-1
uUuuuccuaccccucccauacc		1	1	JCK-3
ucuuuccuaccccucccauacU		1	1	LT-1
ucuuuccuacGccucccauacc		3	1	JCK-3
ucuuuccuaccccucccauCcc		1	1	JCK-1
ucuuucUuaccccucccauacc		1	1	JCK-1
ucuuuccuaccccucccGuacc		1	1	JCK-1
ucuuuccuaccccucccauacc		226	0	LT-2
ucuuuccuaccccucccauacc		2190	0	JCK-2
ucuuuccuaccccucccauacc		1673	0	JCK-3
ucuuuccuacUccucccauacc		114	1	JT-1
ucuuuccuaccccucccauacc		26	0	LCK-1
ucuuuccuacUccucccauacc		1	1	JCK-2
ucuuuccuaccccucccauacc		18	0	JT-3
ucuuuccuacGccucccauacc		2	1	JCK-2
ucuuuccuaccccucccCuacc		6	1	JCK-2
ucuuuccuaccNcucccauacc		3	1	JCK-1
ucuuuccuaccccuccUauacc		1	1	JCK-2
ucuuuccuaccccucccauaccU		6	1	JCK-1
ucuuuccuaccccucccauacca		2	0	LT-3
ucuuuccuaccccucccauacca		1	0	JCK-1
ucuuuccuaccccucccauacca		1	0	JCK-2
ucuuuccuaccccucccauaccU		2	1	JCK-2
ucuuuccuaccccucccauaccU		2	1	JCK-3
ucuuuccuaccccucccauacca		1	0	LT-1
ucuuuccuaccccucccauaccU		11	1	LT-1
ucuuuccuaccccucccauaccU		5	1	LT-3
ucuuuccuaccccucccauaccUu		6	1	LCK-3
ucuuuccuaccccucccauaccaA		3	1	LT-2
ucuuuccuaccccucccauaccUu		2	1	LT-2
ucuuuccuaccccucccauaccUuu		3	1	LT-2
ucuuuccuaccccucccauaccUuu		1	1	LT-3
ucuuuccuaccccucccauaccaCuc		1	1	JCK-2
ucuuuccuaccccucccauaccauuG		1	1	LT-1
cuuuccuaccccucccau		2	0	LCK-3
cuuuccuaccccucccau		1	0	JCK-3
cuuuccuaccccucccaua		4	0	LCK-3
cuuuccuaccccucccaua		1	0	JCK-3
cuuuccuaccccucccaua		1	0	JCK-1
cuuuccuaccccucccauacc		11	0	JCK-1
cuuuccuaccccucccauacc		11	0	JCK-2
cuuuccuaccccucccauacc		16	0	JCK-3
cuuuccuaccccucccauacc		6	0	LT-3
cuuuccuaccccucccauaccauuU		2	1	JCK-2
cuuuccuaccccucccauaccauuU		2	1	JCK-3
uuuccuaccccucccaua		4	0	LCK-3
uuuccuaccccucccauU		1	1	LT-3
uuuccuaccccucccaua		1	0	LT-1
uuuccuaccccucccaua		1	0	LT-2
uuuccuaccccucccaua		5	0	JCK-3
uuuccuaccccucccaua		2	0	LT-3
uuuccuaccccucccaua		1	0	JCK-2
uuuccuCccccucccauacc		2	1	LT-3
uuuccuaccccucccauacc		33	0	LT-1
uuuccuaccccucccauacU		1	1	LT-1
uuuccuaccccucccauacc		8	0	LT-2
uuuccuaccccucccauacc		10	0	JCK-3

```
                                                                                                                                  Star                                                                        Mature
uuccuuuuuuucaccaaaaucuagacucaauuaauuucucuuuucccuuccuccaccaucugguguauuucuuuucucacuaaaaggauggaagagggaaaggcuuaagaguguggguggaagugauagguaugggggguuaggacagagagaaauacucucuuuuccccaauuuauuuuuuuuaauuggggggaagggaaugaauucuuuccuaccccucccauaccauucauuuccauccuuuccuuc
................................................................................................................................................................................................................uuuccuaccccucccauacc......................  28  0  LT-3
................................................................................................................................................................................................................uuuccuaccccucccauacc......................  18  0  JCK-2
................................................................................................................................................................................................................Cuuccuaccccucccauacc......................  2  1  LT-2
................................................................................................................................................................................................................uuuccuaccccucccauacc......................  11  0  LCK-3
................................................................................................................................................................................................................uuuccuaccccucccauacc......................  12  0  JCK-1
................................................................................................................................................................................................................uuuccuaccccucccauacca.....................  2  0  JCK-3
................................................................................................................................................................................................................uuuccuaccccucccauacca.....................  1  0  JCK-2
................................................................................................................................................................................................................uuuccuaccccucccauacca.....................  2  0  LT-1
................................................................................................................................................................................................................uuuccuaccccucccauacca.....................  1  0  JCK-1
................................................................................................................................................................................................................uuuccuaccccucccauaccU.....................  2  1  LT-1
................................................................................................................................................................................................................uuuccuaccccucccauaccU.....................  1  1  JCK-3
................................................................................................................................................................................................................uuuccuaccccucccauacca.....................  3  0  LCK-3
................................................................................................................................................................................................................uuuccuaccccucccauaccU.....................  1  1  JCK-2
................................................................................................................................................................................................................uuuccuaccccucccauacca.....................  1  0  LT-3
................................................................................................................................................................................................................uuuccuaccccucccauaccU.....................  1  1  JCK-1
................................................................................................................................................................................................................uuuccuaccccucccauacca.....................  2  0  JT-1
................................................................................................................................................................................................................uuuccuaccccucccauaccau....................  10  0  JCK-1
................................................................................................................................................................................................................uuuccuaccccucccauaccau....................  2  0  LCK-3
................................................................................................................................................................................................................uuuccuaccccucccauaccau....................  5  0  JT-2
................................................................................................................................................................................................................uuuccuaccccucccauaccau....................  4  0  LT-1
................................................................................................................................................................................................................uNuccuaccccucccauaccau....................  1  1  JCK-2
................................................................................................................................................................................................................uuuccuaccccucccauaccUu....................  1  1  LT-1
................................................................................................................................................................................................................uuuccuaccccucccauaccau....................  15  0  JCK-2
................................................................................................................................................................................................................uuuccuacccAucccauaccau....................  1  1  JCK-2
................................................................................................................................................................................................................uuuccuaccccucccauaccau....................  6  0  JT-3
................................................................................................................................................................................................................uuuccuaccccucccauaccau....................  7  0  LT-3
................................................................................................................................................................................................................uuuccuaccccucccauaccau....................  7  0  JCK-3
................................................................................................................................................................................................................uuuccuaccccucccauaccUuu...................  2  1  LT-2
................................................................................................................................................................................................................uuuccuaccccucccauaccauu...................  3  0  LT-1
................................................................................................................................................................................................................uuuccuaccccNcccauaccauu...................  1  1  JCK-1
................................................................................................................................................................................................................uuuccuaccccucccauaccauu...................  2  0  JCK-3
................................................................................................................................................................................................................uuuccuaccccucccauaccauu...................  3  0  JCK-2
................................................................................................................................................................................................................uuuccuaccccucccauaccauu...................  2  0  LT-2
................................................................................................................................................................................................................uuuccuaccccucccauaccauu...................  3  0  LT-3
................................................................................................................................................................................................................uuuccuacccGucccauaccauu...................  1  1  LT-2
................................................................................................................................................................................................................uuuccuaccccucccauaccauuc..................  3  0  LT-1
................................................................................................................................................................................................................uuuccuaccccucccauaccauuc..................  3  0  JCK-2
................................................................................................................................................................................................................uuuccuaccccucccauaccauuc..................  1  0  JCK-1
................................................................................................................................................................................................................uuuccuaccccucccauaccauuc..................  6  0  JCK-3
................................................................................................................................................................................................................uuuccuaccccucccauaccauuc..................  1  0  LCK-3
................................................................................................................................................................................................................uuuccuaccccucccauaccauuc..................  1  0  LT-3
................................................................................................................................................................................................................uuuccuaccccucccauaccauuU..................  1  1  LT-1
.................................................................................................................................................................................................................uuccuaccccucccauacc......................  3  0  LT-1
.................................................................................................................................................................................................................uuccuaccccucccauacc......................  1  0  LT-3
.................................................................................................................................................................................................................uuccuaccccucccauacc......................  8  0  JT-1
.................................................................................................................................................................................................................uuccuaccccucccauacc......................  6  0  LCK-3
.................................................................................................................................................................................................................uuccuaccccucccauaccauu...................  2  0  JCK-3
.................................................................................................................................................................................................................uuccuaccccucccauaccauu...................  5  0  LCK-3
.................................................................................................................................................................................................................uuccuaccccucccauaccauu...................  2  0  LT-1
.................................................................................................................................................................................................................uAccuaccccucccauaccauu...................  2  1  JCK-1
.................................................................................................................................................................................................................uuccuaccccucccauaccauu...................  1  0  JCK-2
.................................................................................................................................................................................................................uAccuaccccucccauaccauu...................  2  1  JCK-3
.................................................................................................................................................................................................................uuccuaccccucccauaccauuU..................  1  1  LT-1
.................................................................................................................................................................................................................uuccuaccccucccauaccauuc..................  4  0  JCK-1
.................................................................................................................................................................................................................uuccuaccccucccauaUcauuc..................  1  1  JCK-2
.................................................................................................................................................................................................................uuccuaccccucccauaccauuc..................  11  0  JCK-2
.................................................................................................................................................................................................................uuccuaccccucccauaccauuc..................  4  0  JCK-3
..................................................................................................................................................................................................................uccuaccccucccauacc......................  2  0  LCK-3
..................................................................................................................................................................................................................uccuaccccucccauacc......................  1  0  LT-2
..................................................................................................................................................................................................................uccuaccccucccauacc......................  3  0  JT-2
..................................................................................................................................................................................................................uccuaccccucccauacc......................  5  0  JT-3
..................................................................................................................................................................................................................uccuaccccucccauacc......................  2  0  LT-3
..................................................................................................................................................................................................................uccuaccccucccauacc......................  1  0  LT-1
..................................................................................................................................................................................................................uccuaccccucccauacc......................  1  0  JCK-2
..................................................................................................................................................................................................................uccuaccccucccauacc......................  1  0  JCK-1
..................................................................................................................................................................................................................uccuaccccucccauacca.....................  5  0  LT-2
..................................................................................................................................................................................................................uccuaccccucccauacca.....................  5  0  JT-3
..................................................................................................................................................................................................................uccuaccccucccauaccauu...................  20  0  LT-1
..................................................................................................................................................................................................................uccuacccAucccauaccauu...................  1  1  JCK-2
..................................................................................................................................................................................................................uccuaccccucccauaccauu...................  85  0  JCK-2
```

Star (uauggggguuaggacagaga) — **Mature** (ucuuuccuaccccucccauacc)

uuccuuuuuuucaccaaaaucuagacucaauuaauuucucuuuucccuuccuccaccaucugguguauuucuuuucucacuaaaaggauggaagagggaaaggcuuaagagugugggguggaagugauagguauggggguuaggacagagagaaauacucucuuuccccaauuuauuuuuuuuaauuggggggaagggaaugaauucuuuccuaccccucccauaccauucauuuccauccuuuccuuc

uNcuaccccucccauaccauu	1	1	JCK-3
uccuaccccucccauaccauu	53	0	JCK-3
uccuaccccucccauaccauu	5	0	LT-2
uccuaccccucccauaccauu	11	0	LT-3
uccuaccccucccauaccauu	52	0	JCK-1
uccuaccccucccaCaccauu	1	1	JCK-2
uccuaccccucccauaccCuu	1	1	JCK-1
uccuaccccucccCuaccauuc	1	1	JCK-2
uccuaccccucccauaccauuc	142	0	JCK-2
uccuaccccucccauaccauuU	3	1	JCK-1
uccuaccccucccauaccauuU	3	1	JCK-3
uccuacccAucccauaccauuc	1	1	JCK-3
uccuaccccucccauaccauuc	5	0	LT-2
uccuaccccucccauaccauuc	66	0	JCK-1
uccuaccccucccauaccauuU	6	1	JCK-2
uccuaccccucccauaccauuc	101	0	JCK-3
uccuaccccuAccauaccauuc	1	1	JCK-1
uccuaccccucccauaccauuc	23	0	LT-3
uccuaccccucccauaccauuU	5	1	JT-3
uccuaccccucccauaccauuc	16	0	LT-1
uccuaccccucccauaccauuc	8	0	LCK-3
uccuaccccucccauaccauuU	1	1	LT-1
uccuaccccucccauCccauuc	1	1	JCK-1
uccuaccccucccauaccauuU	2	1	LT-3
uccuaccccucccauaccauuU	3	1	JT-2
uccuCcccucccauaccauuc	1	1	JCK-2
uccuaccccucccauaccauuA	1	1	JCK-3
Cccuaccccucccauaccauuc	1	1	JCK-2
ccuaccccucccauacca	2	0	LT-2
ccuaccccucccauacca	1	0	LT-3
ccuaccccucccauaccau	3	0	JCK-3
ccuaccccucccauaccauu	4	0	LT-2
ccuaccccucccauaccauu	5	0	JCK-1
ccuaccccucccauaccauu	20	0	JCK-2
cUuaccccucccauaccauu	1	1	JCK-2
ccuaccccucccauaccauu	1	0	LCK-3
ccuaccccucccauaccauu	12	0	JCK-3
ccuaccccucccauaccauu	3	0	LT-3
ccuaccccucccauaccauu	3	0	LT-1
ccuaccccucccauaccauuU	1	1	LT-3
ccuaccccucccauaccauuc	4	0	LT-1
ccuaccccucccauaccauuc	22	0	JCK-1
ccuaccccucccauaccauuU	5	1	JCK-3
ccuaccccCcccauaccauuc	1	1	JCK-2
ccuaccccucccauaccauuc	5	0	LT-3
ccuaAcccucccauaccauuc	1	1	JCK-1
ccuaccccucccauaccauuc	32	0	JCK-3
ccuaccccucccauaccauuU	2	1	JCK-1
ccuaccccucccauaccauuU	5	1	JCK-2
ccuaccccucccauaccauuc	34	0	JCK-2
ccuaccccucccauaccauucU	3	1	LT-2
ccuaccccucccauaccauuca	1	0	JCK-1
cuaccccucccauaccau	3	0	LT-1
cuaccccucccauaccauu	6	0	LT-1
cuaccccucccauaccauu	5	0	LT-3
cuaccccucccauaccaCu	1	1	LT-2
cuaccccucccauaccauu	3	0	JCK-2
cuaccccucccauaccauu	4	0	LCK-3
Uuaccccucccauaccauu	1	1	LT-1
cuaccccucccauaccauu	1	0	LT-2
cuaccccucccauaccauuU	2	1	JCK-3
cuaccccucccauaccauuU	1	1	LT-2
cuaccccucccauaccauuc	5	0	LT-2
cuaccccucccauaccauuc	4	0	LT-1
cuaccccucccauaccauuU	1	1	LT-1
cuaccccucccauaccauuU	1	1	JCK-2
cuaccccucccauaccauuc	3	0	JCK-3
cuaccccucccauaccauuc	5	0	JCK-2
cuaccccucccauaccauuU	3	1	LCK-3
cuaccccucccauaccauuc	6	0	LT-3

```
Star        Mature
uuccuuuuuuucaccaaaaucuagacucaauuaauuucucuuuucccuuccuccaccaucugguguauuucuuuucucacuaaaaggauggaagagggaaaggcuuaagagugugggugggaagugauagguauggggguuaggacagagagaaauacucucuuuuccccaauuuauuuuuuuuaauugggggaagggaaugaauucuuuccuaccccucccauaccauucauuuccauccuuuccuuc
...cuaccccucccauaccauuc.................  3  0  JCK-1
...cuaNcccucccauaccauuc.................  1  1  JCK-1
...cuCccccucccauaccauuc.................  1  1  LT-3
...cuaccccucccauaccauucU................  1  1  LCK-3
...cuaccccucccauaccauuca................  1  0  LT-1
...cuaccccucccauaccauuca................  2  0  JCK-1
...uaccccucccauaccauuc.................  1  0  LT-1
...ccauaccauucauuuccauccu.......  2  0  LT-1
...ccauaccauucauuuccauccuAu.....  1  1  JCK-2
...ccauaccauucauuuccauccuAu.....  1  1  JCK-3
...cauaccauucauuuccauc.........  1  0  LT-1
...cauaccauucauuuccauU.........  1  1  JCK-2
...cauaccauucauuuccauccu.......  1  0  LT-3
...cauaccauucauuuccauccuu......  2  0  JCK-3
...cauaccauucauuuccauccuu......  1  0  JCK-2
...cauaccauucauuuccauccuuu.....  2  0  JCK-3
...cauaccauucauuuccauccuuu.....  1  0  JCK-2
...cauaccauucauuuccauccuuuc....  2  0  JCK-1
...auaccauucauuuccauc.........  1  0  LT-3
...auaccauucauuuccauccuu......  1  0  LT-3
...auaccauucauuuccauccuuu.....  1  0  LT-3
...auaccauucauuuccauccuuuc....  1  0  LT-1
...auaccauucauuuccauccuuuc....  1  0  LT-2
...auaccauucauuuccauccuuuU....  1  1  JCK-3
...auaccauucauuuccauccuuucc...  3  0  JCK-2
...auaccauucauuuccCuccuuucc...  1  1  JCK-2
...auaccauucauuuccauccuuuccuu.  1  0  LT-3
...uaccauucauuuccauccuuucc...  2  0  JCK-1
...auucauuuccauccuuucc...  2  0  LT-3
...auucauuuccauccuuuccu..  2  0  LT-2
...auucauuuccauccuuuccuu.  3  0  LT-3
```
